# How harassment is depriving universities of talent: a national survey of STEM academics in the UK

**DOI:** 10.3389/fpsyg.2023.1212545

**Published:** 2024-01-29

**Authors:** Lukas F. Litzellachner, Julie Barnett, Lucy Yeomans, Leda Blackwood

**Affiliations:** Department of Psychology, University of Bath, Bath, United Kingdom

**Keywords:** gender, workplace harassment, retention, STEM – science technology engineering mathematics, academia

## Abstract

**Introduction:**

Despite efforts to increase girls’ interest in subjects related to science, technology, engineering, and mathematics (STEM) careers, there remains a large gender gap in STEM academic faculty.

**Methods:**

We conducted a national survey comprising 732 early career and senior academics from 40 universities in the UK to investigate the role of pull (receiving career advancement opportunities) and push (experiencing harassment) factors in shaping people’s intentions to stay in STEM academia, and the mediating role of perceived workplace climate, academic identification, and beliefs about the ability to succeed (job-related self-efficacy).

**Results:**

Our findings show the differential effect of harassment experiences for women, relative to men. Women experienced more harassment than men, which contributes to their higher intentions to leave academia through enhancing perceptions of a negative workplace climate (i.e., a less collaborative, fair, and inclusive climate) and lower job-related identification (i.e., believing in their ability to succeed as researchers). While receiving opportunities also related to intentions of leaving academia, we did not observe a gender difference in this factor.

**Discussion:**

The result of our analysis underlines the critical importance of preventing and addressing harassment in academic institutions for the retention of female academic talent.

## Introduction

Universities in the UK and beyond have set their sights on improving gender diversity of faculty; particularly within STEM fields (science, technology, engineering, and mathematics). Pointing to the scale of the problem in the UK, the Institute of Physics (2017) reported that only 19% of staff in physics departments were female, with skewed gender ratios also existing in disciplines such as chemistry (30% women), mathematics (27% women), and engineering (15% women). A contributing factor to this imbalance is that women are more likely to leave academic positions than men (Geuna and Shibayama, 2015; Cech and Blair-Loy, 2019). While providing equal opportunities to all employees should be every employer’s goal, universities have more than one reason for why they should care about the exodus of female academic staff. Research has found a lack of diverse representation in faculty positions to be detrimental to key educational outcomes such as staff-student relations (Mercer-Mapstone et al., 2021), student achievement (Cross and Carman, 2021), and the quality of scientific output (Freeman and Huang, 2014). Furthermore, the lack of visible female role models in the workforce contributes to the erroneous perception that STEM jobs are fundamentally masculine (Makarova et al., 2019), causing the inequality to become self-perpetuating as fewer non-male candidates apply to fill vacancies in the field. Therefore, creating an environment where women scientists are comfortable with remaining in academia is crucial to breaking this vicious circle.

**Figure 1:**
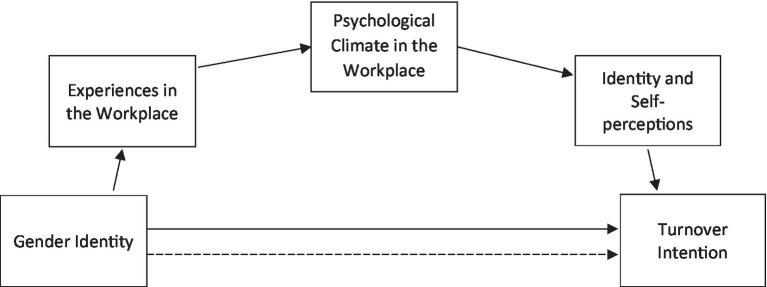
Working model of the influence of gender identity on turnover intention.

The question of why women leave STEM academia in greater numbers than men and so where to focus policy interventions is a pressing one. Research points to the importance of structural issues such as the employment of early career researchers on short-term contracts; and experiences in the workplace such as high workloads and poor work-life balance, and systemic biases affecting career trajectories (e.g., selection and promotion processes; publication and research funding; Miller et al., 2014). The premise of our research is that a critical proximal factor in the decision to stay in an academic career – particularly in a competitive and demanding context – is the strength of a person’s academic identity. In accordance with the social identity perspective (Tajfel et al., 1971; Turner, 1987) we argue that entailed in academic identity is a sense of belonging and alignment with academic values and behaviours, which in turn is consequential for one’s sense of agency and ability to succeed. Importantly, however, research on marginalised or peripheral group members shows that the identification process is not straightforward. Rather, it depends on being recognised by other members of the group – particularly those deemed prototypical such as senior academics (Ellemers et al., 2013). From this perspective, one’s experiences in the workplace – including those identified above – are important in part because they signal how we are regarded and whether we can see ourselves as valued (and valuable) members of the group (Tyler et al., 1996). Accordingly, in this study, we adopt the working model seen in Figure 1. As there exists no study that simultaneously investigates how experiences, psychological workplace climate perceptions, and self-perceptions are interrelated with academic staff turnover, the current investigation does the important work of empirically connecting all the individual pieces of our model. However, before doing so, it is important to examine the empirical evidence that already exists for the relationships specified in the model.

According to Social Identity Theory (Tajfel et al., 1971) and Self Categorization Theory (Turner, 1987), identification with one’s work-related groups (e.g., profession, organisation, team) plays an important role in guiding organisational behaviours (Greco et al., 2021). When we identify with an important and contextually salient group, we assimilate to the normative beliefs, values, and behaviours of the group (that is, we self-stereotype). Because we derive self-esteem from our group memberships, we are motivated to see our group in positive terms, thus we experience greater trust, cooperate more with colleagues, and strive to enhance both the group’s reputation and our own reputation within the group (Ellemers, 2001; Tyler, 2001; Janssen and Huang, 2008). According to the Group Engagement Model (Tyler and Blader, 2003) which is informed by the social identity perspective, the quality of interactions with other group members is key to the identity process. Thus, for women working in male-dominated fields (such as STEM academia) positive experiences in the workplace are found to be critical to a host of positive workplace outcomes (Karanika-Murray et al., 2015; Hameed et al., 2021), including retention (van Dick et al., 2006).

Research on psychological climate, defined as an individual’s perception of organisational structures, processes, and events (Parker et al., 2003), identifies a range of areas that are important to people’s organisational experience and commitment. Following the advice of McKay et al. (2007), we examine psychological climate from a holistic perspective, including perceptions that might be particularly important for female faculty’s workplace outcomes. Therefore, we specifically focus on perceptions that are identified in research on collaborative climate (Riffle et al., 2013), diversity climate (McKay et al., 2007), and fairness climate (Colquitt et al., 2002; Simons and Roberson, 2003).

First, in a collaborate climate, organisational members support each other’s performance, aid each other in their development, share knowledge, and build positive relationships (Chen and Huang, 2007). The source of a collaborative climate are experiences of support from colleagues and higher-ups, and the absence of uncivil behaviour (Agervold and Mikkelsen, 2004; Ho et al., 2018). As we would expect, individuals who perceive a more collaborative climate report a greater feeling of organisational identification (Kuruppuge et al., 2018) and confidence to succeed in the job (Erdwins et al., 2001; McCabe et al., 2017; Zhu et al., 2019), as their self-perceptions as organisational members are validated. In turn, a collaborative climate contributes to a significantly lower turnover rate (McNatt and Judge, 2008; Kumar and Singh, 2012). The opposite of a collaborative climate is a competitive climate, in which organisational members believe they have to compete with colleagues for resources and opportunities (Fritz and van Knippenberg, 2017). Competitive climates have been linked to increases in felt ostracism in the workforce, and future turnover (Ng, 2017). There is also some evidence that competitive climates put women at an additional disadvantage; competitive norms grant additional incentive for majority members to use their power and exclude minority members in order to maximise the ingroup-outgroup difference for a boost in their collective self-esteem (Haslam, 2001; Eagly and Karau, 2002; Fritz and van Knippenberg, 2017). These findings, coupled with the general perception of academia as a competitive (e.g., *publish-or-perish*) work environment (Carson et al., 2013), make employee perceptions of a collaborative climate especially relevant for our investigation of female faculty turnover.

Second, diversity climate refers to perceptions, attitudes, and beliefs about how the organisation and its members relate to people from underrepresented backgrounds (Mor Barak et al., 1998). Employees in a positive diversity climate theoretically experience less conflict between their personal identity and their work-related social identity because people from all walks of life can be seen as prototypical members of the organisation. Conceptually, then, a good diversity climate will emerge from experiences of inclusive practises (i.e., equal distribution of opportunities) and a lack of discrimination (Shore et al., 2011). Empirically, diversity climate is also positively related to identification with the organisation and better workplace inclusion (Hofhuis et al., 2016), as well as increased psychological capital (i.e., a set of job resources including confidence to succeed on the job; Newman et al., 2018). Similar to the findings for a positive collaborative climate, there is evidence for perceptions of a positive diversity climate being important for all groups of employees, but particularly important for women and ethnic minority employees (McKay et al., 2007). For instance, Parker et al. (1997) found the association between organisational support for equal opportunity and / or affirmative action perceptions of fair outcomes and processes (organisational justice) and career development opportunities was especially strong for these groups.

Lastly, fairness climate refers to the perception that people in the work environment are treated fairly in terms of both outcomes (distributive justice) and processes (procedural justice: Tyler, 1989; Colquitt et al., 2002). A climate of fairness (or organisational justice) originates from the top; when the actions of management are perceived as fair, the risk of conflict is reduced (through perceived neutrality), organisational trust is boosted, and the value of employees as organisational members is affirmed. Showcasing the importance of perceived fairness, a low climate of fairness is related to perceptions of a lack of opportunities, especially when these opportunities seem unfairly distributed based on (for example) gender stereotyping (Whisenant et al., 2015). Likewise, harassment experiences also lower the perception of a just climate (especially when not dealt with by the organisation), because harassment inherently and unfairly advantages some members of the organisation at the cost of others (Rubino et al., 2018). Accordingly, fairness climate (including both distributive and procedural justice) which has been associated with organisational identification (cf. Chen et al., 2015), and perceived internal job resources such as self-efficacy (Elshaw, 2010), may be especially important for creating better workplace outcomes for women (Fields et al., 2000).

Perceptions of psychological climate are ultimately based in people’s experiences interacting with others. In general terms, such workplace experiences can be either pull factors or push factors. Pull factors are pleasant experiences that create an inviting, positive psychological climate, affirm people’s identification, and their value as academics; attracting them to remain in their academic careers. Push factors are unpleasant experiences that create a hostile, negative psychological climate, highlighting interpersonal (and intergroup) differences and ostracising some academics, ultimately deterring them from remaining in their academic careers.

By the above definition, a prominent pull factor should be receiving support and opportunities for career development. In fact, a lack of opportunities for development and progress is among the most cited reasons for career exit amongst academics (Stroh et al., 1996; Downes et al., 2014; Porter et al., 2019). Faculty positions in many STEM subjects continue to be predominantly occupied by men who, as a group, may be motivated by ingroup bias or the threat to status that women entering their field represents (Tajfel and Turner, 1979; Haslam, 2001). Certainly, compared to their male colleagues, women receive fewer opportunities to co-author research papers (Belle et al., 2014; Ozel et al., 2014; Frances et al., 2020); less recognition for their work in the form of prizes and research funding (Lincoln et al., 2012; Witteman et al., 2019); and their support networks are less likely to include men in higher positions (Spurk et al., 2015). Being excluded from opportunities for career development by senior colleagues (e.g., supervisors) has been found to be negatively associated with perceptions of a positive, cooperative climate (Ho et al., 2018). Thus, we assume experiences of opportunity to be important pull factors for (female) academics.

While receiving opportunities for career advancement should pull people into staying in academia, experiences of harassment can push people out of academia (Roberts and Ayre, 2002; Settles et al., 2007). Harassment includes any type of behaviour that pressures, provokes, frightens, intimidates, humiliates or demeans a person (Berdahl, 2007). Whilst some forms of harassment such as ‘*quid pro quo*’ demands for sexual favours rely on unequal status relations within an organisation, other forms that create a hostile environment (e.g., offensive jokes) do not, and may even be performed by lower status individuals (Maass et al., 2013). Women are more commonly the victims of harassment compared to men and are at greater risk where they are vulnerable (e.g., due to age, ethnicity, or career stage; Berdahl and Moore, 2006; Stockdale and Nadler, 2012) or violate gender role expectations (Berdahl, 2007). Similar to the withdrawal of support and opportunities, harassment is often committed out of a desire to dominate female colleagues, whose high occupational status threatens the presumptive superiority of men in the field (Berdahl, 2007; McLaughlin et al., 2012).

The prevalence of harassment has been shown to have a detrimental impact on physical and mental health (Cantisano et al., 2008), self-perception and self-stereotyping (Hundhammer and Mussweiler, 2012), cognitive performance (Gay and Castano, 2010), and organisational commitment (Schneider et al., 1997). For instance, Aycock et al. (2019) found that harassment undermined female physics students’ self-confidence and belonging at their workplace. Moreover, there is evidence that harassment impacts not just those who are the direct targets; women (and men) who witness harassment learn something about who is and who is not valued and included within an organisation (Dhanani and LaPalme, 2019). Thus, Bradley-Geist et al. (2015) showed that vicariously experiencing sexism in the workplace, negatively impacted female’s self-esteem and career aspirations.

In terms of organisational climate, frequent harassment incidences have also been linked to employee perceptions of a less inclusive, more competitive, and less just climate (Agervold and Mikkelsen, 2004). It is worth noting that this association might go in both directions; studies have found that a competitive climate also breeds harassment (Salin, 2003; Sischka et al., 2020). Moreover, a critical aspect of harassment is how its handling by the organisation affects the psychological climate. Frequent harassment that goes unpunished can result in perceptions of the organisation being negligent toward or permissive of hostile behaviour (Cortina and Areguin, 2021). Thus, there is an inherent link between the prevalence of harassment and a perception of the organisational climate as negative, competitive, and unfair. Crucially, harassment has been shown to negatively affect employee mental health and workplace outcomes both directly and via negative organisational climate perceptions (Richman et al., 1999; Barankay, 2010), and to constitute an important push factor for (female) academics (Gim et al., 2015; National Academies of Sciences, Engineering, and Medicine, 2018).

The focus of this research is on the kinds of experiences (positive and negative) that have been linked to turnover intentions of female staff, where there is potential for policy intervention. We examine both the direct effects of these experiences on turnover intentions as well as the potential mediating psychological processes. In line with our working model (Figure 1), we expect that self-perceptions are the most proximal predictor of the intention to leave academia, followed by psychological climate perceptions, and lastly self-reports of experiences. We believe that, by mapping out the process, our findings can inform actionable policy interventions that maximise the amount of talent staying in academia.

To answer the questions related to these aims, we used a quantitative cross-sectional questionnaire study with a large sample of STEM academics from 40 universities across the United Kingdom. We hypothesised that there would be a mediational process represented in our working model, where the association of an experiential variable (e.g., received opportunities, harassment experiences) with the intention to still work in STEM in academia in 5 years is mediated by both a workplace perception (e.g., perceiving a cooperative, diversity friendly, and fair work climate) and a self-perception (e.g., identifying as an academic and career confidence and commitment). However, due to the novelty of our design we were agnostic as to the specific combination of experiences, psychological climate perceptions, and self-perceptions that would best predict the intentions of (female) academic staff to stay in academia.

## Method

### Sample

We recruited 835 faculty members from 40 universities in the United Kingdom (UK) via our networks within UK STEM departments. Participants were drawn from various STEM departments, including biological science (18%), computer science (7%), engineering (28%) mathematical science (16%), and physics (13%). We excluded 23 respondents who had no variance across their answers (either because they did not submit answers to the questionnaire or because they gave the same answer to all questions). We also made a deliberate decision to exclude 6 respondents who self-categorised in terms of a career path that was not primarily academic (e.g., technician). Because we were primarily interested in why people leave academia, but not why people stop being researchers, we excluded 74 respondents who indicated that they would stop doing research both inside and outside of academia within 5 years. The final sample of 732 respondents (280 cis-women, 328 cis-men, 27 non-cis people, 97 prefer not to say; *M_age_* = 34.41, *SD_age_* = 10.48) was comprised of 288 PhD-Students, 123 Post-Docs, 71 Fellows (research and teaching fellows), 75 Lecturers, and 148 senior academics (senior lecturers, assistant professors, and full professors), with 27 preferring not to say their current career stage. Most respondents were employed on fixed-term contracts (61%), with only 33% on more secure, open ended contracts. Additionally, most respondents identified as white (70%; non-white: 14%), straight (69%; non-straight: 13%), with 8% having disability status (vs 75% not having disability status). Most respondents also reported that their parents had themselves obtained a higher education degree (university degree: 57%; no university degree: 28%).

### Materials

The questionnaire was designed in collaboration with university staff involved in the UK’s Athena Swan framework to advance gender equality in higher education. Accordingly, some items were drawn from institutional surveys and were assumed to have face validity. An effort was made to ensure that theoretically informed items drawn from social psychological literature had ecological validity in this context. Unless stated otherwise, respondents answered questions on a 7-point Likert-scale ranging from “Strongly disagree” (coded 1) to “Strongly agree” (coded 7).

### Self-perceptions

#### STEM academic identity

We operationalised STEM academic identity as a multifaceted construct drawing on frameworks by Cameron and Lalonde (2001), Leach et al. (2008), Kernis and Goldman (2006), Schmader and Sedikides (2018), and Tyler and Blader (2003). We asked participants to indicate agreement with 10 statements measuring *centrality* (e.g., “Being an academic is an important part of my self-concept”), *similarity / prototypicality* (e.g., “I have a lot in common with the average academic,” “I am typical of most academics”), *belonging* (e.g., “I feel a strong sense of belonging as an academic,” “I sometimes feel on the periphery as an academic”), *recognition* (e.g., “Sometimes I think other academics doubt my credentials,” “I am considered to have academic expertise,” “Other academics recognise me as a valued member”), and *authenticity* (e.g., “I sometimes hide aspects of my identity to avoid negative attention from other academics,” “I can always be myself around other academics”). For the final academic identity score, we took the mean of the responses to all 10 items (*M* = 4.35, *SD* = 0.96).

#### Career self-perceptions

We measured our participants’ STEM academic career perceptions through two items drawn from Blau’s (1989) career commitment scale (“I like this career too much to give it up,” and “If I could do it all over again, I would choose to work in a different profession”) and three modified items from Cook et al. (1981) measure capturing career-related self-efficacy or confidence to succeed (“Someone like me can succeed in an academic career,” “I am satisfied with my chances for getting ahead in an academic career,” “Sometimes I doubt whether I will succeed in an academic career”) (reversed), (*M* = 4.27, *SD* = 1.15).

### Workplace diversity and inclusion perceptions

Our measure of workplace perceptions drew on the organisational climate literature described above and included adapted and self-constructed measures of important dimensions of diversity and inclusion in an academic setting: collegiality and inclusion in the workplace; perceived diversity and fair and equal treatment; and bias in recruitment and promotions. To assess respondents’ perceptions of *collegiality and inclusion* in the workplace, respondents were asked how much they agreed or disagreed with eight statements. Example items include “There is a spirit of collegiality and support” and “Social activities (e.g., parties, team building) are welcoming to all” (*M* = 4.68, *SD* = 0.99).

We measured *support for diversity and fair treatment* with seven items, asking respondents to agree (or disagree) with statements about how they see people being treated at their workplace. Example items include: “There are diverse role models from diverse backgrounds,” and “People with different backgrounds are treated fairly” (*M* = 4.26, *SD* = 1.18).

Finally, three items specifically asked about *bias in recruitment and promotion* processes (“From my observation, there is bias in academic promotion processes,” “Academic recruitment processes select the best person for the job,” and “From my observations, there is bias in academic recruitment processes”) (*M* = 3.37, *SD* = 1.20).

Because they were theoretically closely related, we conducted an exploratory factor analysis to see whether we were justified in combining the three scales measuring workplace perceptions. The analysis suggested a two-factor solution, with the first factor explaining 26% of variance and the second factor explaining 16% of variance. However, there was considerable cross loading between these two factors, which ended up being highly correlated, *r* = 0.54, *p* < 0.001. Thus, to stay true to our conceptual model, we still combined all items into one measure assessing workplace perceptions (*M* = 4.28, *SD* = 0.95).

### Experiences

#### Received opportunities

To indicate whether they felt like they had received opportunities for career development in academia, respondents were asked to answer six items in comparison to others at their career stage. Example items include “I have opportunities to collaborate on publications” and “I have opportunities to build professional networks” (*M* = 5.16, *SD* = 1.15).

#### Harassment experiences

Respondents were asked to report how often they had experienced or witnessed insulting or offensive remarks or behaviours at work. The five items that were given addressed harassment based on (1) religion, (2) gender or sexuality, (3) socio-economic background, (4) disability, and (5) ethnicity. Each item was responded to twice, once for whether the respondent had experienced the harassment themselves, and once for whether the respondent had witnessed someone else being harassed. For all items, respondents could indicate that they had experienced this kind of harassment either “Never” (1), “Once” (2), “More than once” (3), or “Frequently” (4). The final harassment score was created by taking the mean of all responses (*M* = 1.29, *SD* = 0.42).

### Outcome measure

#### Staying in academia

We asked respondents where they saw themselves in terms of their career in 5 years. Respondents were given three response options: “Not working in a STEM field,” “Working in a STEM field, but NOT in academia,” and “Working in a STEM field in academia.” After removing all individuals who would not be working in STEM at all (mainly due to retirement), the resulting variable was dichotomous, with 460 respondents reporting the intent to stay in academia and 272 respondents reporting the intent to leave academia within 5 years.

### Control variables

#### Affective workplace climate evaluation

We included a sematic differential measure, asking respondents to affectively evaluate their workplace along 8 dimensions (safe-intimidated, angry-calm, accepted-rejected, included-excluded, disappointed-pleased, happy-sad, hopeful-hopeless, insecure-confident). For each dimension, respondents evaluated their workplace on a scale from 1 to 7. Before combining the individual ratings for each respondent, we recoded all answers so that high scores always represent positive evaluations (*M* = 4.85, *SD* = 1.26).

#### Collaborative working style

Our model holds that self-perceptions should be related to environmental perceptions, not individual working patterns. Controlling for collaborative work preferences (a self-perception) ensures that any effects of positive climate are due to perceptions of the culture of behaviour in the workplace. Thus, we added a section about working style, that contained four items related to collaborative working preferences, such as “I prefer to work independently and alone” (inverse), “I thrive in a competitive environment” (inverse),” “I prefer to work in collaboration with others,” and “I feel comfortable working in a group” (*M* = 5.10, *SD* = 0.99).

### Procedure

Respondents completed an online survey in which details about their employment were collected at the beginning and additional demographic information was collected at the end. The middle section of the survey contained the study variables in the following order: identity and career perceptions; staying in academia; collaborative working style, received opportunities; workplace diversity and inclusion and affective workplace climate; and experience of harassment. The order of the individual items within each block was randomised across participants. Open questions were also included throughout the survey; responses to these questions provide additional confidence in the validity of our measures and our findings and are reported elsewhere (Corbett et al., under review).^1^

### Transparency and openness

We have detailed our sampling plan and all data preparation (i.e., exclusion criteria), alongside descriptions of all measures in the method section above. All data, the analysis code, and the research materials are available at Blackwood and Litzellachner (2023). Data were analysed using R version 4.1.2. The study’s design and analyses were not preregistered.

## Results

Table 1 shows correlations among all variables in the analysis. All continuous variables were z-transformed to obtain standardised coefficients in regression. To account for the considerable skew in the age distribution of our sample, our models only include the natural logarithm of age. Because the correlations within the same class of variables (e.g., psychological climate perceptions) were considerably large (e.g., between collaborative climate and diversity climate), we inserted all variables into one model first to assess their variance inflation factor (VIF). Commonly, a VIF larger than 2.5 suggest cause of concern, even though smaller VIF values have been shown to relate to spurious findings (Johnston et al., 2018). Concerning the demographic variables, a χ^2^ tests of independence showed a strong association between career stage and the nature of the respondent’s contract (*χ^2^*(4) = 514.49, *p* < 0.001, *r* = 0.85), while a spearman correlation showed a strong relationship between career stage and age (*r_s_* = 0.83, *p* < 0.001). As the outcome had the strongest relationship with career stage amongst these variables (*χ^2^*(4) = 151, *p* < 0.001, *r* = 0.46; compared to with contract type: *χ^2^*(4) = 107.1, *p* < 0.001, *r* = 0.39; and age: biserial *r* = 0.37, *p* < 0.001), contract type and age were omitted from the main analyses. Furthermore, Positive Workplace Climate also showed high collinearity (VIF = 3.84). To solve this issue, we merged the Positive Workplace Climate perceptions with the Affective Workplace Evaluations. This choice was made due to the high correlation that existed between the two variables. While resulting score had great internal consistency (*α* = 0.94, *M* = 4.3, SD = 0.96), combining the scores did not completely resolve the multicollinearity problem regarding the positive workplace climate perceptions (remaining VIF = 2.57). However, this variable is now only strongly correlated with predictors from other levels, which we (*a-priori*) theorised to not have a parallel relationship with the outcome, but to influence the outcome both directly and indirectly through each other. Thus, the residual collinearity might contain information about mediated effects and should not be removed (Johnston et al., 2018).

**Table 1:** Correlations between all continuous variables in the analysis and the DV.

	(1)	(2)	(3)	(4)	(5)	(6)	(7)
Received opportunity	[0.82]						
Harassment experiences	−0.12**	[0.82]					
Identifying as an academic	0.47***	−0.26***	[0.80]				
Positive workplace climate	0.40***	−0.46***	0.41***	[0.90]			
Career self-perceptions	0.46***	−0.18***	0.61***	0.46***	[0.73]		
Affective workplace evaluation	0.44***	−0.38***	0.50***	0.74***	0.54***	[0.92]	
Collaborative style	0.19***	0.05	0.13**	−0.00	0.01	0.15***	[0.78]
DV	0.25***	0.05	0.33**	0.05	0.43***	0.16***	−0.02

All correlations with the DV (Staying in Academia) are point-biserial correlations where positive coefficients signify a higher likelihood of staying. Values in brackets on the diagonal represent Cronbach’s alpha. ^t^*p* < 0.1, *p < 0.05, **p < 0.01, ***p < 0.001.

Due to the multilevel nature of the data (academics from different universities), we tested the assumption of independence of observations using intraclass correlation (ICC). Not accounting for a multilevel structure in the data can lead to distortions in the analysis (Musca et al., 2011). Intraclass correlation coefficients were reliably low (ICC(1) = 0.05), justifying the use of single-level analysis methods. Logistic regression was chosen, due to the dichotomous nature of the outcome variable.

### What predicts staying in academia?

Before our main analysis, we conducted a chi square test and replicated findings for women reporting higher intentions to leave academia *χ^2^*(1) = 5.64, *p* = 0.018. We then conducted hierarchical logistic regression including first all demographic variables, then adding all experiences, then all climate experiences, and lastly all self-perceptions (see Table 2). We observed an effect of career stage; fellows (*β* = 1.72, *p* < 0.001, *OR* = 6.08), lecturers (*β* = 2.48, *p* < 0.001, *OR* = 10.91), and senior academics (*β* = 2.89, *p* < 0.001, *OR* = 15.56) were all significantly more likely to indicate an intention to stay in academia than PhD-Students. However, there was no difference between early career-researchers; post-docs were not significantly more likely to express an intention to stay than PhD-Students (*β* = 0.18, *p* = 0.458, *OR* = 1.14). These effects of career stage remained highly significant throughout all models. When positive (received opportunities) and negative (harassment) experiences were included at the second step, received opportunities was significantly related to a higher likelihood of staying (*β* = 0.54, *p* < 0.001, *OR* = 1.72), but experiences of harassment were not (*β* = 0.11, *p* = 0.416, *OR* = 1.12). At the third step, perceiving a diverse and inclusive climate significantly related to the likelihood of staying in STEM academia (*β* = 0.56, *p* = 0.002, *OR* = 1.75). But with this measure of workplace climate in the model, personal experiences of received opportunities no longer contributed (*β* = 0.27, *p* = 0.095, *OR* = 1.31) and experiences of harassment became a significant predictor (*β* = 0.37, *p* = 0.026, *OR* = 1.44). Lastly, when STEM academic identity and career self-perceptions were included, there was no significant effect of identifying as an academic (*β* = 0.03, *p* = 0.891, *OR* = 1.03) but having confidence to succeed in a STEM academic career was significant (*β* = 1.06, *p* < 0.001, *OR* = 2.89). At this final step in the model, experiences of harassment remained a significant positive predictor of the intention to stay (*β* = 0.34, *p* = 0.048, *OR* = 1.40), but the perception of a diverse and inclusive workplace had lost its positive effect on intending to stay (*β* = 0.08, *p* = 0.722, *OR* = 1.08).

**Table 2:** Standardised log-odds ratio coefficients of the hierarchical logistic regression analysis.

	Model 1	Model 2	Model 3	Model 4
*Demographics*				
Career stage (vs. PhD-student)				
Post-doc	0.07	−0.11	0.20	0.46
Fellow	1.72***	1.81***	2.16***	2.08***
Lecturer	2.48***	2.39***	2.79***	2.71***
Senior academic	2.89***	2.74***	3.16***	2.67***
Gender (vs. cis-men)				
Cis-women	−0.18	−0.30	−0.27	−0.19
Non-cis people	−0.37	1.43	1.03	0.32
Non-heterosexual (vs. heterosexual)	0.09	0.18	0.15	0.14
Parents without higher education (vs. parents with higher education)	0.05	−0.19	−0.22	−0.18
Non-white ethnicity (vs. white ethnicity)	0.37	0.27	0.47	0.53
Disability (vs. no disability)	−0.08	0.02	−0.13	−0.04
*Experiences*				
Received opportunity		0.54***	0.27^t^	0.10
Harassment experience		0.11	0.37*	0.34*
*Psychological climate perceptions*				
Positive workplace climate			0.56**	0.08
*Self-perception*				
Identifying as an academic				0.03
Career self-perceptions				1.06***
Collaborative working style^a^				−0.20

^a^Control variable. ^t^*p* < 0.1, *p < 0.05, **p < 0.01, ***p < 0.001.

### Mediation analysis

The pattern of findings in our hierarchical logistic regression analysis hints at the plausibility of a full-mediational pathway from received opportunity experiences to intentions to stay through diverse and inclusive workplace perceptions and career self-perceptions. However, this does not preclude the existence of a separate pathway through harassment experiences. Hence, we wanted to explore this possibility via a mediation analysis. We included gender as the predictor variable to explore the possibility of this mechanism affecting cis-women more than cis-men.

Because our mediation analysis involves both continuous and dichotomous variables as outcomes in regression analysis (requiring both OLS and logistic regression), we used bootstrapping to test for the significance of the indirect effect. For this procedure, we ran 5,000 iterations with randomly drawn sub-samples (with replacement) of equal size to the original, calculating the indirect effect for each sub-sample. We simultaneously tested the indirect effects for both kinds of experiences. Thus, for each of the two indirect effects, a three-mediator model was calculated as laid out by Hayes’s (2013) model 6 (equation 1):


(1) $$\mathrm{IndirectEffec}t_{k}=a_{1k}d_{21k}d_{32}b_{3}$$

where *a_1_* is the effect of the initial predictor (gender) on the first mediator (received opportunities or harassment experiences), where the subscript *k* is 1 for received opportunities and 2 for harassment experiences, *d_21_* is the effect of the first mediator on the second mediator (diverse and inclusive climate), controlling for the initial predictor, *d_32_* is the effect of the second mediator on third mediator (career self-perceptions) controlling for the first mediator and the initial predictor, and *b_3_* is the effect of the third mediator on the outcome (the likelihood to stay in academia in 5 years). The remaining variables from the hierarchical regression were retained as control variables whenever a coefficient for the model was estimated. To make them comparable, all effect sizes were converted to Pearson’s *r* from partial eta-squared (OLS regression) and odds-ratios (OR; logistic regression) using the effect-size conversion guidelines by Borenstein et al. (2009). For the indirect effect to be considered significant, more than 4,875 out of 5,000 simulations (i.e., 97.5%) needed to show a positive indirect effect.

**Figure 2:**
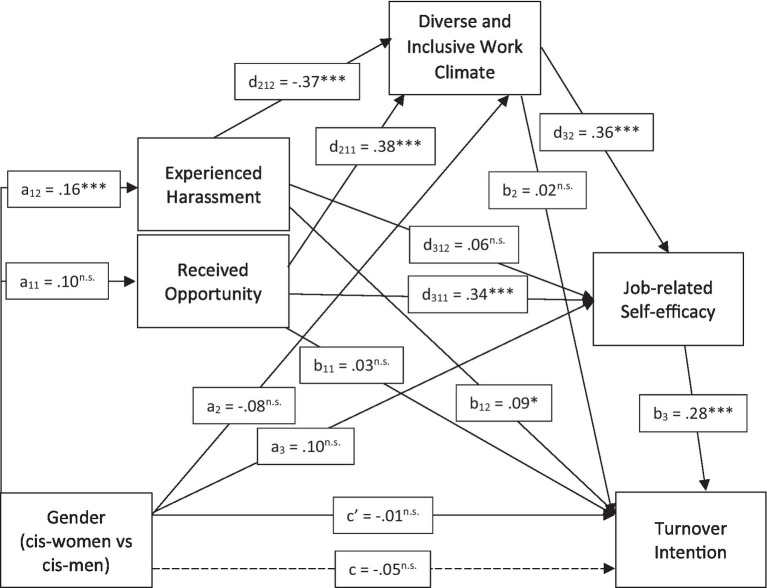
Mediation model from gender to turnover intention. ^t^*p* < 0.1, *p < 0.05, **p < 0.01, ***p < 0.001.

Figure 2 shows the results of our mediational bootstrapping. First, we observed no direct effect of gender on the intention to leave when all control variables were included. However, the possibility of mediation is still given, as the absence of a direct effect does not preclude the presence of an indirect effect (Rucker et al., 2011). Therefore, we investigated the indirect effects through both received opportunities and harassment experiences via bootstrapping the result of equation 1. We observed no significant indirect effect of gender via receiving opportunities ($a_{11}d_{211}d_{32}b_{3}$ = 0.00, *p* = 0.287; i.e., 4,283 out of the 5,000 simulated samples showed a positive indirect effect). However, there was an independent significant indirect effect of gender on the intention to leave academia through harassment experiences, diverse and inclusive workplace climate, and career self-perceptions ($a_{12}d_{212}d_{32}b_{3}$ = −0.01, *p* < 0.001; i.e., 5,000 out of the 5,000 simulated samples showed a negative indirect effect). Thus, we conclude that differences in retention between male and female academic staff are more likely due to the more frequent harassment experiences that women face, rather than because they receive fewer opportunities than men. It should be mentioned that received opportunities themselves had a significant and independent indirect effect on turnover intentions via the same mediators as harassment experiences (diverse and inclusive climate and career self-perceptions; $d_{211}d_{32}b_{3}$ = 0.02, *p* < 0.001; i.e., 4,998 out of the 5,000 simulated samples showed a positive indirect effect). However, because we did not observe differences between women and men in terms of received opportunities, only the difference in harassment experiences is likely to explain why more women than men exit academic careers.

## Discussion

More women than men leave academia for comparable jobs in industry (Geuna and Shibayama, 2015; Cech and Blair-Loy, 2019). To guide academic policy and facilitate interventions, we explored a model that views this leaving behaviour from a social identity perspective. We expected that perceptions of identity and career self-perceptions (confidence to succeed) were crucial to leaving decisions, and grounded in perceptions of the psychological workplace climate, which ultimately root in specific frequent pull and push experiences – being granted opportunities and experiencing harassment –in the academic context. Our large sample of STEM academics from 40 different UK Universities confirmed the plausibility of this model. Our results show that career self-perception was the most proximate and strongest predictor of intending to stay in academia. This self-perception was enhanced in a climate of diversity and inclusivity, which was in turn independently influenced by experiences of opportunity (positively) and experiences of harassment (negatively). Thus, our study grants actionable guidance to policy makers and practitioners on what experiences are likely increasing the retention of academic staff, while making critical contributions to psychological theory about the relationship between identity processes and workplace climate.

While both experiences in our analyses were independent and opposite forces that predicted staying in academia, only one of the two – experiences of harassment – was more likely to occur for cis-women (compared to cis-men). Further, our mediational analysis confirmed that gender does have an indirect connection to the intention to leave through experiences of harassment. This is not surprising, as harassment is very prevalent on university campuses, not just among students (Yoon et al., 2010), but also among staff (Vargas et al., 2020), and is more likely to affect women than men (Zurbrügg and Miner, 2016).

As we predicted, the experience of harassment was related to the intention to leave academia, via its effects on the perception of the workplace climate as diverse and inclusive, and career self-perceptions (having confidence to succeed). Surprisingly, we also found a residual positive direct effect of harassment on the intention to leave academia. This finding suggests that, when harassment experiences do not negatively influence the workplace climate, they link to increased likelihood of staying. This finding hints at the importance of correctly handling a harassment incident. An organisation that fails to handle what is often a very powerful negative experience appropriately (that is, take action rather than passively acknowledge, refrain, or deny), will receive less trust from its victimised employees, while an organisation that acts decisively and swiftly may inspire trust in victimised employees (Chrobot-Mason and Hepworth, 2002).

It is important to note that our harassment measure included various types of harassment (based on religion, gender/sexuality, socioeconomic background, disability, and ethnicity), both self-experienced and witnessed being done to others. The fact that all these items correlate well enough to justify combining them is in line with research on differences between majority and minority groups in perceptions of harassment (McCord et al., 2018) and in perspective taking more generally (Galinsky 2002; Demoulin et al., 2009). Therefore, our findings call academic employers to act conclusively to prevent harassment in their work environments.

Although the effects of receiving opportunities on staying in academia were independent of gender, they highlighted an important theoretical point about the importance of environmental variables in shaping employees’ workplace commitment and confidence to succeed, captured by career self-perceptions. This is consistent with the notion that such self-perceptions are not an enduring quality of the person but rather, contextual and malleable as per Bandura’s (1994) conceptualisation of self-efficacy and current theorising informed by the social identity perspective (Haslam, 2001). Therefore, our findings not only urge practitioners and policy makers to nurture confidence to succeed in the academic workforce, but also provide actionable guidance on how to do so (by providing opportunities and preventing harassment).

In line with our model, we found a strong partial mediation effect of positive workplace climate perceptions between both kinds of experiences and the academic career self-perceptions. As predicted by the group-value model (Tyler et al., 1996), perceiving a positive interpersonal climate in the workplace led to higher identification with the occupation (as seen in the zero-order correlations; Table 1). This raises the question as to why did we not observe significant effects of identifying as an academic on the intention to remain in academia? Akin to the causality issues around the direct effect of harassment, this might have been a consequence of the cross-sectional nature of our design. As there is no way to identify the causal strength of the connections between the variables, there are potentially other models consisting of some of these variables that might also provide a good fit to the data. Many of these configurations, in fact, do show a significant effect of identification as an academic. For example, academic identity was a significant predictor of intention to stay if entered before the climate perceptions and career self-perceptions (*β* = 0.49, *p* = 0.003, *OR* = 1.64). As this effect existed independently of the effect of received opportunity (*β* = 0.37, *p* = 0.013, *OR* = 1.45), which was the dominant effect in our model, it potentially constitutes another pathway to a positive diversity climate.

Future research should consider mixed methods approaches including longitudinal designs to investigate the causal pathways and experimental and qualitative designs to investigate the underlying theorised processes in our model. Whilst our focus has been on explaining women’s attrition from STEM academia, our findings raise important questions about men and women’s sensitivities to and perceptions of harassment of women in the workplace. Just as it is important to understand how and why harassment might undermine women’s confidence in an academic future, it is equally important to understand the effects on men who may be either perpetrators or allies. There is evidence to suggest that perceiving pervasive harassment emboldens would be perpetrators (Maass et al., 2013); and this may be sustained by ingroup bias leading to majority group members failing to recognise harm (Greenwald and Pettigrew, 2014). By the same token, perceiving harassment by fellow ingroup members may be experienced as a threat to identity and there is some evidence for misogyny within the workplace negatively impacting men (Miner-Rubino and Cortina, 2007). Moreover, we cannot assume that trust in organisations will be universally bolstered by support for victims.

A clear implication of our findings for institutions of higher education is the need to focus on harassment as a determining factor in the attrition of women from STEM academia. An understanding of harassment as an intergroup process draws attention to the complex and dynamic motivational causes and consequences; this understanding is important to knowing where and how to intervene. A recent systematic review (Bondestam and Lundqvist, 2020) points to the lack of rigorous evidence for the long-term effectiveness of most conventional harassment prevention strategies (e.g., organisational policy, harassment education or training, case management, or victim support structures). Thus, the critical task of future research is to find effective theoretically informed and evidence-based intervention methods.

## Conclusion

To address the exodus of female academics we conducted research which provides evidence for the practical value of preventing harassment in academic workplaces for the prevention of voluntary turnover. We found that female academics were especially negatively affected by experiences of harassment, which harmed their view of their psychological workplace climate, and their job-related self-perception, which finally increased their likelihood to report intentions of leaving academia. As intentions to leave were especially prominent among early career researchers (i.e., PhD-Students), our research highlights the importance of positive work environment full of advancement opportunities, especially at these stages. Ultimately, we believe that decisive action from academic decision-makers on the opportunities for improvement identified by our research can help provide an academic work environment in which female talent does not feel the need to leave but is instead empowered to flourish and thrive.

## Data availability statement

The dataset analysed for this study is available in the University of Bath Research Data Archive. https://doi.org/10.15125/BATH-01271.

## Ethics statement

The studies involving humans were approved by Psychology Research Ethics Committee, University of Bath. The studies were conducted in accordance with the local legislation and institutional requirements. The participants provided their written informed consent to participate in this study.

## Author contributions

JB and LB: conceptualized. LB: designed the study. LY: data collection. LL: data analyses and visualizations. LL and LB: wrote original draft and wrote revised draft. JB and LY: commented on drafts. All authors contributed to the article and approved the submitted version.

## Footnotes

^1^Corbett, E., Barnett, J., Yeomans, L., and Blackwood, L. *"That’s just the way it is": bullying and harassment in STEM academia. [Manuscript submitted for publication].*
